# Novel cost-effective method of laparoscopic feeding-jejunostomy

**DOI:** 10.4103/0972-9941.55108

**Published:** 2009

**Authors:** Rajesh C Mistry, Sanket S Mehta, George Karimundackal, C S Pramesh

**Affiliations:** Department of Thoracic Surgery, Tata Memorial Hospital, Mumbai, India

**Keywords:** Laparoscopic, feeding-jejunostomy, minimal-access, feeding procedure

## Abstract

A feeding jejunostomy tube placement is required for entral feeding in a variety of clinical scenarios. It offers an advantage over gastrostomies by eliminating the risk of aspiration. Standard described laparoscopic methods require special instrumentation and expensive custom-made tubes. We describe a simple cost-effective method of feeding jejunostomy using regular laparoscopic instruments and an inexpensive readily available tube. The average operating time was 35 min. We had no intra-operative complications and only one post-operative complication in the form of extra-peritoneal leakage of feeds due to a damaged tube. No complications were encountered while pulling out the tubes after an average period of 5–6 weeks.

## INTRODUCTION

Enteral feeding has several advantages over parenteral feeding. A feeding jejunostomy tube placement is required for enteral feeding in a variety of clinical scenarios. It offers an advantage over gastrostomies by eliminating the risk of aspiration. Minimal access methods have been described for the placement of feeding jejunal tubes, but they often require special equipment not routinely available in all operation theatres.[1–3] Also, most of these methods require specially designed feeding tubes which are expensive and not easily available.[4–6] We describe an indigenous method for totally laparoscopic feeding jejunostomy tube placement using simple laparoscopic instruments and an inexpensive, readily available feeding tube.

## METHOD

**Figure 1:**
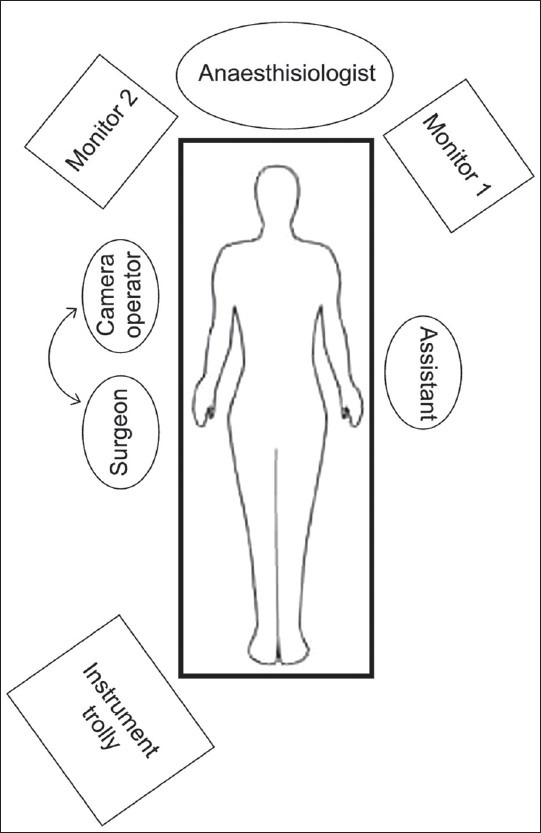
The operating room set-up

**Figure 2:**
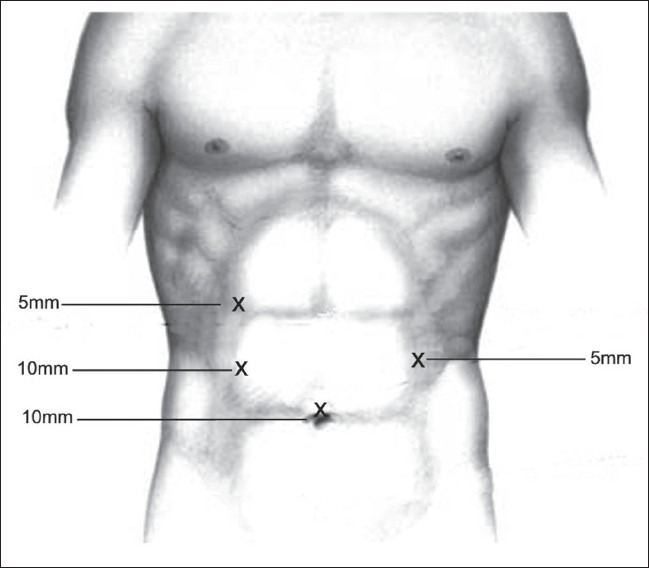
Port sites

No specific pre-operative preparation of the patient is required for this procedure. The operating room (OR) setup is as shown in the illustration in Figure 1. The patient is placed in the supine position and the port sites are as shown in the illustration in Figure 2. The additional monitor for the assistant is optional. The procedure was conceptualized as an adjunct to surgery for cancer of the oesophagus for which laparoscopic stomach mobilization and stomach tube preparation are done. Hence, the position of the patient, the operating room setup and the port positions are as would be required for laparoscopic mobilization of the stomach. However, we found that the same port positions provided an advantage while doing a laparoscopic feeding jejunostomy.

**Figure 3:**
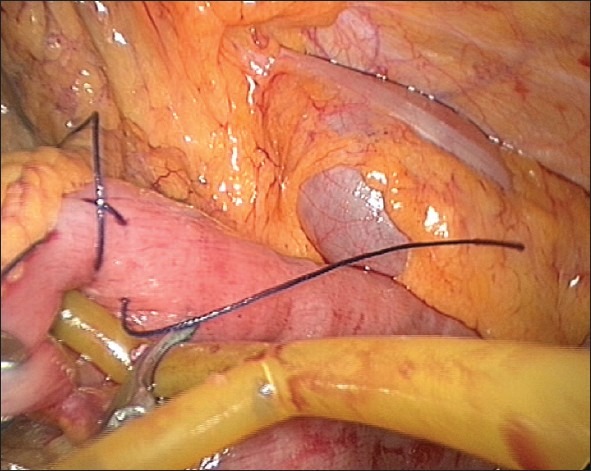
Insertion of T-tube in the jejunal opening

**Figure 4:**
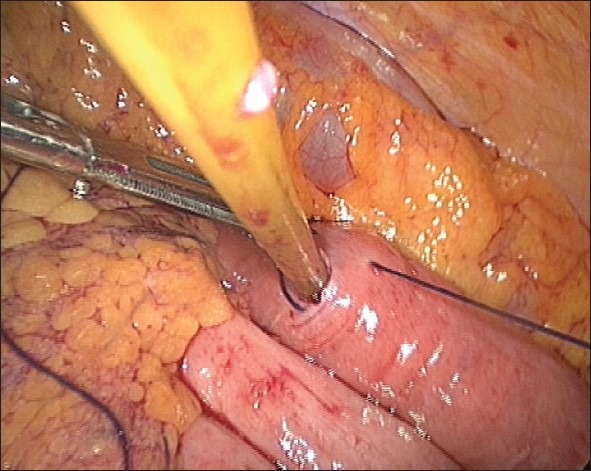
Completed insertion of the horizontal limbs of the T-tube in the jejunum and the sero-muscular purse-string stitch

**Figure 5:**
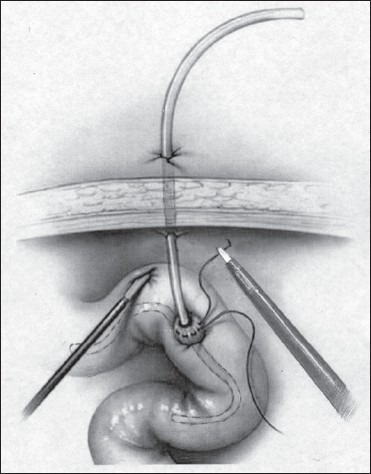
Sero-muscular purse-string stitch (illustration)

**Figure 6:**
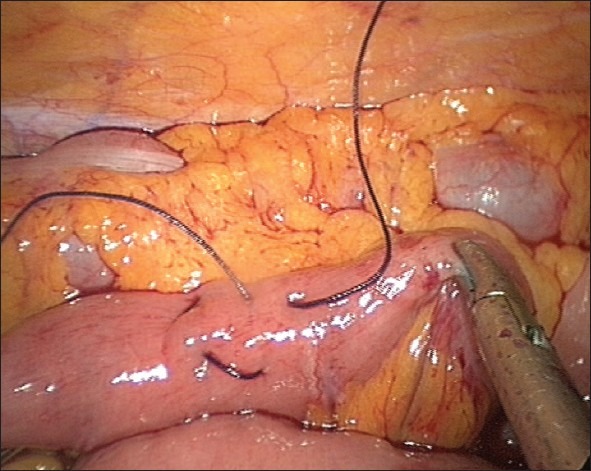
Sero-muscular purse-string stitch taken prior to insertion of the T-tube

**Figure 7:**
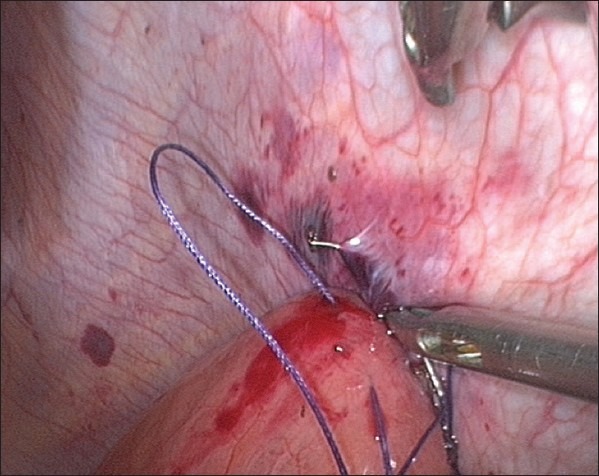
Stitches taken to hitch the jejunostomy exit site to the anterior abdominal wall (proximal stitch)

**Figure 8:**
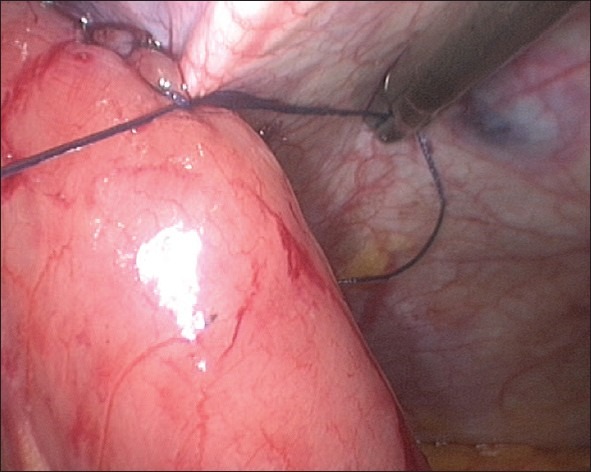
Stitches taken to hitch the jejunostomy exit site to the anterior abdominal wall (distal stitch)

**Figure 9:**
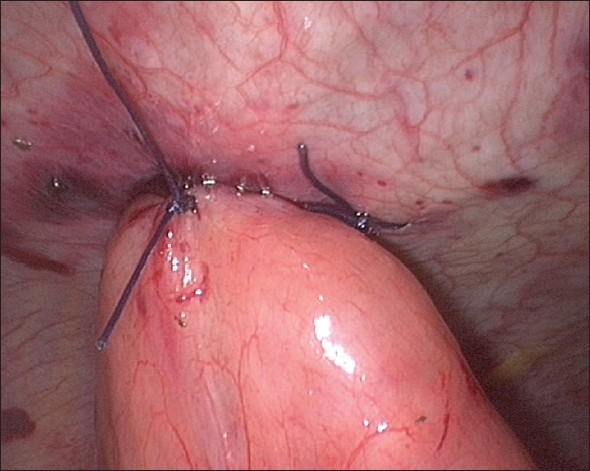
Stitches taken to hitch the jejunostomy exit site to the anterior abdominal wall (completed final picture)

We prefer to get intraperitoneal access by the open method at the umbilical port site. We insert the remaining ports under vision, and the left-sided port site also serves later as the exit of the feeding tube. We find it easier to initially identify the duodenojejunal flexure and the proximal jejunum with the camera in the umbilical port. A bowel grasper introduced through the left paramedian port helps to lift the colon; bowel graspers introduced through the two right-sided ports are then used to identify the duodenojejunal flexure. Once the loop is identified, a point approximately 40 cm from the duodenojejunal flexure is selected as the jejunostomy site. We then pass a grasping forceps from the right lower paramedian port to the left-sided port and through the port outside. Once the grasper has passed through the port outside, the left paramedian port is removed. The grasper is then used to hold the feeding tube and deliver it into the peritoneal cavity. We use a simple 12-Fr T-tube, with the horizontal limbs cut to 4-cm length each, as the feeding tube. The choice of this tube was based on our experiences with the other custom-made feeding tubes that were expensive, not easily available and difficult to introduce and hitch. Also, they are of a smaller calibre making them liable to blockage with regular feeds.[7] The simple T-tube is easily available, inexpensive, of adequate calibre, easy to introduce and because of its T-joint, is self-retaining and easy to hitch laparoscopically. Once the horizontal limb of the T-tube is within the peritoneal cavity, the camera is then shifted to the right lower paramedian port, and the umbilical and the right upper paramedian ports are used for instrumentation. An opening is then made at the selected site of the proximal jejunal loop using monopolar diathermy attached to a closed Maryland grasper. The two horizontal limbs of the T-tube are then introduced into the jejunum one after the other [Figure 3]. As the limbs are equal in length, there is no worry as to the direction of introduction or the orientation of the afferent and the efferent loops during the introduction of the tube. Also, as the T-joint is the site of exit of the tube from the intestine, there is no concern regarding the adequacy of the intra-intestinal length of the tube, in contrast to the other custom-made tubes that need measuring the intrajejunal length and introduction specifically in the efferent direction for proper placement. After the two limbs are in position, a seromuscular purse-string suture is placed at the exit site of the tube using an absorbable suture material, as shown in the illustration in Figures 4,5. Alternatively, the purse-string suture can be taken before creating the jejunal opening and tied once the tube is in place [Figure 6]. The integrity of this stitch is checked by pushing a dilute betadine solution through the T-tube and looking for any leak. A gentle tug on the tube from the outside then gets the loop up to the site of exit from the abdominal wall. After confirming the orientation of the afferent and the efferent loops, two seromuscular sutures are taken and hitched to the abdominal wall, one on the proximal and one on the distal side of the purse-string stitch to decrease the tension at the site of exit of the tube [Figures 7–9]. A third stitch is taken on the skin exit site to hitch the tube in slight traction. The port sites are then closed taking adequate precaution to close the sheath for the 11-mm ports. Feeding through the tube can start after 12 h.

## RESULTS

We performed the procedure in 19 patients undergoing oesophageal resection from May to November 2008. Using this method, we had no intra-operative complications related to the procedure. The average time required for the procedure was 35 min. Post-operatively, we had no port-site complications, and feeding was started for all patients on the first post-operative day. The only complication encountered was extraperitoneal leakage of feeds in one patient. There was no intraperitoneal leak as confirmed by a fluoroscopic conray study. Damage to the vertical limb of the T-tube was the probable cause. The tube was removed on the fifth post-operative day without any further complications. There were no complications faced while removing the tubes after an average period of 5–6 weeks. During the first few cases, we had used the portex T-tube that tends to become stiff with time. Although we had no complications while removing this tube, we did face difficulty in pulling out the tube in one of the cases that required the tube to be kept for a longer time for feeding purposes. This made us switch over to the latex T-tube that is softer and more pliable. We did not have any difficulty in removing the tube thereafter.

## CONCLUSION

Feeding jejunostomy is frequently performed either as an isolated procedure by itself or as an adjunct to specialized surgery like oesophageal and gastric resections and after pancreaticoduodenectomy. Conventionally performed laparoscopic jejunal tube placement requires special equipment, feeding tubes and specially formulated feed solutions. Our innovative method of totally laparoscopic jejunal tube placement is easy, inexpensive, does not require specialized equipment or feeding tubes and is a simple alternative to conventional laparoscopic feeding tube placement.
